# Prenatal exposure to vanadium and lead and its impact on the risk of congenital heart defects in neonates: evidence from the Lanzhou Birth Cohort

**DOI:** 10.3389/fpubh.2026.1758836

**Published:** 2026-03-17

**Authors:** Ying Wei, Jianhao Sun, Zhenzhen Wu, Liangsen Teng, Jie Huang, Xinjuan Jiao, Yun Dang, Xiaoli Zhao, Ying Zhang, Shumei Tuo, Baohong Mao, Qing Liu

**Affiliations:** 1 Gansu Provincial Maternity and Child-care Hospital, Lanzhou, Gansu, China; 2 Institute of Translational Medicine, Medical College, Yangzhou University, Yangzhou, China; 3 Clinical Medical College of Youjiang Medical University for Nationalities, Baise, China; 4 School of Nursing, Gansu University of Chinese Medicine, Lanzhou, China; 5 Qingyang Second People's Hospital, Qingyang, China

**Keywords:** congenital heart defects, lead, maternal blood, pregnancy, vanadium

## Abstract

**Background:**

Using data from the Lanzhou Birth Cohort in China, this study examined the associations between prenatal exposure to vanadium (V) and lead (Pb)—both individually and jointly—and the risk of neonatal congenital heart defects (CHDs).

**Methods:**

This birth cohort study, conducted in Lanzhou, China, included 97 mother-newborn pairs assigned to the case group and 194 pairs serving as controls (1:2 ratio). Maternal blood concentrations of V and Pb were quantified using inductively coupled plasma mass spectrometry. Multivariate logistic regression was used to assess associations between prenatal V and Pb exposure levels and the risk of neonatal CHDs and specific subtypes. Interaction effects were further assessed using both additive and multiplicative models.

**Results:**

Maternal blood V concentrations were positively correlated with Pb levels. Higher maternal blood V levels were significantly associated with an increased risk of CHDs in offspring (*p* = 0.005), including isolated CHDs (*p* = 0.025), multiple CHDs (*p* = 0.013), patent ductus arteriosus (PDA, *p* = 0.025), and atrial septal defects (ASDs, *p* = 0.003). Similarly, elevated maternal blood Pb levels were linked to a higher risk of CHDs (*p* < 0.001), encompassing isolated CHDs (*p* = 0.002), multiple CHDs (*p* = 0.002), PDA (*p* < 0.001), ASDs (*p* = 0.048), and ventricular septal defects (VSDs, *p* = 0.015). Combined prenatal exposure to elevated V and Pb levels demonstrated a significant association with CHDs risk (*p* < 0.001).

**Conclusions:**

Maternal whole blood levels of V and Pb in late pregnancy, whether considered individually or jointly, were significantly associated with an increased risk of CHDs in the offspring.

## 1 Introduction

Congenital heart defects (CHDs) are defined as structural malformations of the heart and/or major blood vessels that are present at birth, arising from disruptions in cardiac development during the fetal period. According to the World Health Organization (WHO), an estimated 1.35 to 1.50 million newborns globally are affected by CHDs annually (1). Despite significant advancements in technology that have enhanced the quality of life and prognosis for patients with CHDs, the condition exerts a lifelong impact, particularly in cases of complex CHDs (2). Consequently, actively identifying and addressing preventable and controllable factors is essential for the prevention of CHDs. Prenatal heavy metal exposure and its impact on offspring development is a prominent topic in perinatal epidemiology. For instance, increased exposure to barium, Lead (Pb), and cadmium has been associated with a higher risk of abortion (3, 4). Similarly, elevated levels of Pb, mercury, and cadmium in pregnant women are correlated with an increased likelihood of preterm birth (5–7). Moreover, prior studies have identified arsenic, cadmium, mercury, cobalt, titanium, and stannum as significant risk factors for CHDs in offspring (8–11).

Vanadium (V), a transition metal and essential raw material, is widely distributed in nature, primarily occurring in compound forms. It plays a critical role in various industries, including steel manufacturing, battery production, aerospace engineering, and medical applications (12). Human exposure to V occurs through environmental and occupational pathways. Environmental exposure is primarily associated with fossil fuel combustion (13), whereas occupational exposure is linked to industries such as fuel-fired power plants, petrochemical production, steel manufacturing, and mining (14). The primary routes of V exposure in humans are respiratory inhalation and gastrointestinal ingestion (15). Animal studies and population-based research have demonstrated that exposure to V and its compounds can adversely impact the digestive, respiratory, nervous, and reproductive systems (16). Previous studies have suggested that prenatal exposure to V is linked to an elevated risk of several adverse birth outcomes, including low birth weight, preterm birth, small for gestational age, and premature rupture of membranes (17–19). However, it remains uncertain whether prenatal exposure to V poses a risk to fetal cardiac development.

Pb is a widely prevalent toxic heavy metal used in various industries, including batteries, automobiles, and dyes, due to industrial advancements (20). Human exposure to Pb primarily occurs through occupational or non-occupational sources, with entry into the body via the respiratory, digestive, or dermal routes (21). In 2021, the U.S. Centers for Disease Control and Prevention (CDC) revised the blood Pb reference value for children aged 1–5 years, lowering the upper limit of exposure from 5.0 μg/dL to 3.5 μg/dL (22). Extensive research has shown that chronic Pb exposure has harmful effects on health, particularly on the cardiovascular, nervous, digestive, urinary, endocrine, and reproductive systems (23). Previous studies have demonstrated that maternal Pb exposure is linked to adverse pregnancy outcomes, including low birth weight, preterm birth, and neural tube defects (6, 24, 25). A case-control study conducted in China found a significant association between elevated maternal hair Pb concentration and an increased risk of CHDs in offspring, including those with CHDs alone and those with CHDs in combination with other defects (26).

Given the limited conclusive research on the relationship between V exposure during pregnancy and offspring cardiac development, and the absence of studies examining the combined effects of V and Pb exposure on the risk of CHDs in offspring, we conducted a prospective nested case-control study in Lanzhou, China. This study aimed to investigate the associations between maternal exposure to V and Pb, both individually and combined, and the risk of CHDs in offspring.

## 2 Materials and methods

### 2.1 Study population

**Figure 1:**
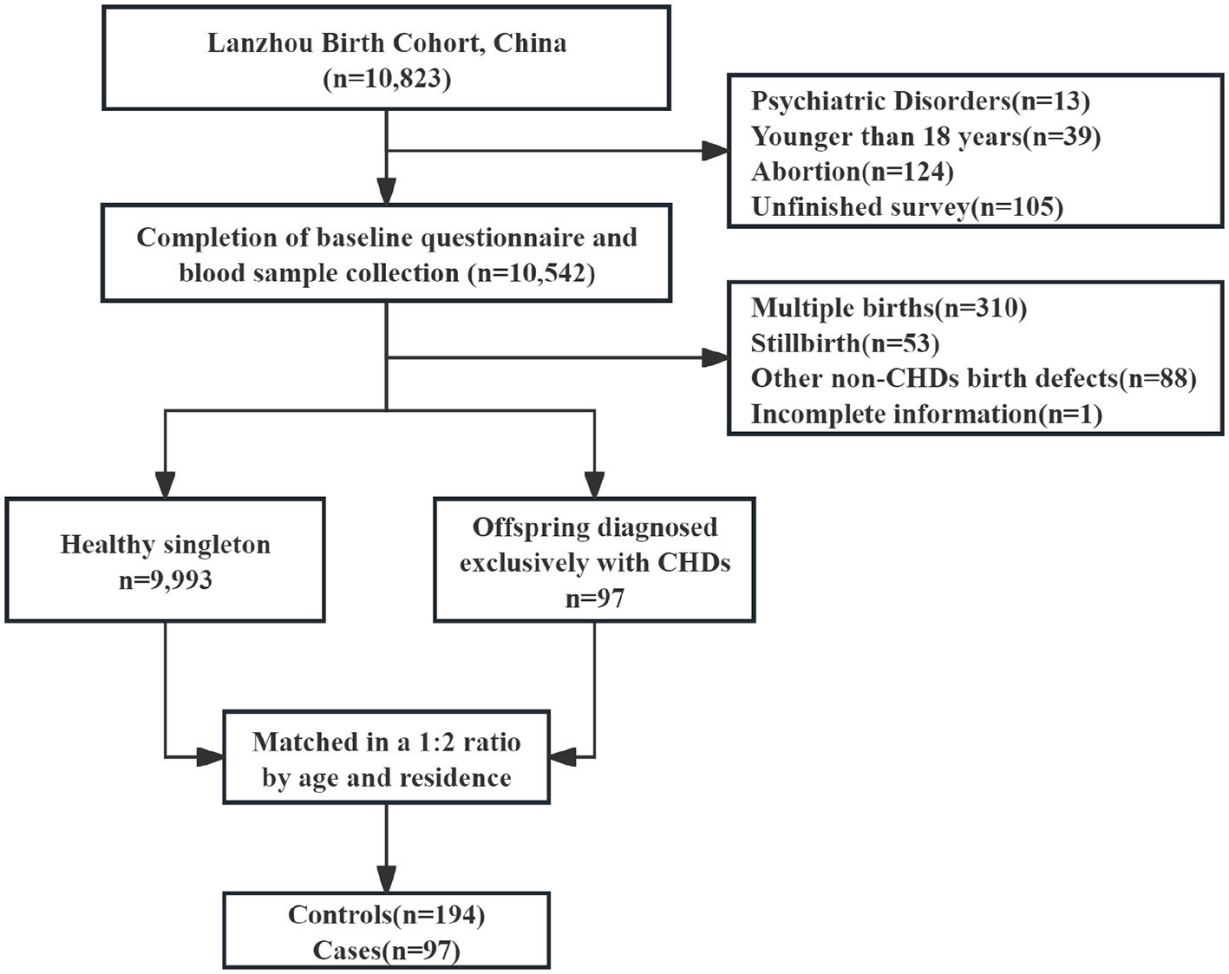
Study population selection flow chart.

A birth cohort study was conducted from 2010 to 2012 at the Gansu Provincial Maternity and Child Care Hospital in Lanzhou, China, involving 10,823 pregnant women (10, 27). After excluding 13 participants with psychiatric disorders, 39 under 18 years of age, 124 who experienced miscarriage before 20 weeks of gestation, and 105 who did not complete the questionnaire, 10,542 participants remained, having completed the baseline questionnaire and provided blood samples. Additional exclusions were made for multiple births, stillbirths, non-CHDs birth defects, and incomplete baseline information. Among the singleton offspring of the final cohort, 97 were diagnosed with CHDs. For this study, mothers who delivered healthy singleton offspring at baseline were selected as the control group, totaling 9,993 participants. A 1:2 random matching process was conducted based on maternal age (±2 years) and place of residence. Ultimately, 291 participants were included in the study (Figure 1). The study was conducted in strict adherence to the ethical standards established by the Declaration of Helsinki and received official ethical clearance from the Research Ethics Committee at Gansu Provincial Maternity and Child Care Hospital (Approval No. 2012-5).

### 2.2 Diagnosis and classification of CHDs

Fetal echocardiography is a widely accessible and frequently employed diagnostic modality for the detection of CHDs (28). The examinations were conducted by senior sonographers with extensive experience, adhering to the guidelines and standards established by the American Society of Echocardiography and the International Society of Ultrasound in Obstetrics and Gynecology (29, 30). Fetal ultrasonography findings were used solely for prenatal screening indications and did not replace postnatal confirmation. The diagnosis of CHDs was ultimately based on postnatal (neonatal period) echocardiography and the International Classification of Diseases, 10th Revision (ICD-10) codes documented in medical records. Each type of CHDs is categorized based on its severity as “isolated,” “multiple,” or “syndromic.” Furthermore, these anomalies were classified using clinical phenotypes and the ICD-10 codes (Q20–Q28). Additional details regarding the classification of CHDs can be found in our previous research (10).

### 2.3 Questionnaire survey and blood collection

After obtaining written informed consent, trained investigators conducted face-to-face interviews with participating pregnant women to administer the Maternal Health Survey Questionnaire within the framework of the China Birth Cohort Study. The following variables were extracted for analysis: maternal age, education level, Han Chinese ethnicity, pregnancy history, parity, receipt of preconception health education, employment during pregnancy, preterm birth, delivery mode, and postpartum blood loss.

Venous whole blood samples (3 mL) were collected from the participants upon hospital admission for delivery (i.e., late pregnancy) by trained phlebotomists into EDTA-containing tubes. Immediately after collection, tubes were gently inverted several times to ensure adequate anticoagulation and then stored at −80 °C. All sample information was recorded in real time in an electronic biobank management system.

### 2.4 Vanadium and lead assessment

The pretreatment of frozen blood samples was performed following the methods outlined in previous studies (11). The concentrations of V and Pb in maternal blood were then determined using inductively coupled plasma mass spectrometry (ICP-MS, ICAP RQ; Thermo Fisher, USA). After tuning the ICP-MS instrument to optimal conditions, and Sc45, Ge7^2^, Rh^103^, and Re^1^87 were selected as internal standards to minimize matrix effects from non-spectral interferences. Each pre-treated sample was analyzed in triplicate, and the results are reported as the mean value. The concentrations of V and Pb in the blood samples were calculated using the following formula:


$$\begin{array}{l} \omega =\rho Vn/m \end{array}$$

Where ω is the mass fraction of V and Pb in the sample (μg/g), ρ is their concentration in the digested sample solution (μg/L), *V* is the final volume of the solution after digestion (mL), *n* is the dilution factor, and *m* is the mass of the sample (g). The limits of detection (LOD) for V and Pb in whole blood were determined to be 0.002 μg/L and 0.008 μg/L, respectively. Concentrations falling below the LOD were assigned a value of zero in the dataset.

### 2.5 Statistical analysis

Data were processed and analyzed using SPSS version 25.0 (IBM, Chicago, USA). Categorical variables are presented as percentages. Univariate analysis between the case and control groups was conducted using chi-square tests for categorical variables. For continuous variables, *t*-tests were used if the data followed a normal distribution, and rank sum tests were employed for those that did not conform to a normal distribution. In the absence of established reference ranges for V and Pb concentrations in maternal blood, this study employed receiver operating characteristic (ROC) curve analysis, guided by methodologies from previous research (31, 32), to determine the optimal thresholds for these concentrations. The optimal critical values for V and Pb in maternal blood were identified as 3.64 μg/L and 86.70 μg/L, respectively. Based on these thresholds, the concentrations of V and Pb in maternal blood were categorized into low concentrations and high concentrations groups. Using low concentrations as the reference, to evaluate the association between prenatal exposure to V and Pb and the risk of CHDs in offspring, odds ratios (ORs) and 95% confidence intervals (CIs) were computed. A *p*-value of less than 0.05 was considered indicative of statistically significant differences. Variables with *p*-values below this threshold in the univariate analysis were subsequently included in the multivariate analysis. Additionally, we utilized both multiplicative and additive interaction models to assess the potential modifying effects of V and Pb on CHDs.

## 3 Results

### 3.1 Characteristics of participants

Of the 97 documented cases of CHDs, 43 were isolated, 48 were multiple, and 6 were syndromic. Among these cases, patent ductus arteriosus (PDA) accounted for 70, atrial septal defects (ASDs) for 48, ventricular septal defects (VSDs) for 7, and atrioventricular septal defects (AVSDs) for 4. Table 1 presents the baseline characteristics of the study participants. Importantly, significant differences were observed between the two groups in terms of premature birth, mode of delivery, postpartum blood loss, maternal blood V concentration, and maternal blood Pb concentration (*p* < 0.05). No significant differences were noted for the other characteristics. The correlation analysis demonstrated a strong positive relationship between maternal blood V and Pb concentrations (*r* = 0.9095, *p* < 0.001).

**Table 1:** Characteristics of the study population.

**Variable**	**Control *N* = 194 (%)**	**Cases *N* = 97 (%)**	***P*-value**
Maternal Age (years)			0.082
≤ 35	174 (89.7)	80 (82.5)	
>35	20 (10.3)	17 (17.5)	
Maternal Education			0.658
Junior high or below	60 (30.9)	34 (35.1)	
Technical secondary school, high school	29 (14.9)	16 (16.5)	
Undergraduate college or above	105 (54.2)	47 (48.4)	
Han Chinese			0.118
Yes	189 (97.4)	90 (92.8)	
No	5 (2.6)	7 (7.2)	
History of Pregnancy			0.863
Yes	70 (36.1)	36 (37.1)	
No	124 (63.9)	61 (62.9)	
History of Childbirth			0.791
Yes	65 (33.5)	31 (32.0)	
No	129 (66.5)	66 (68.0)	
Preconception Health Education			0.559
Yes	83 (42.8)	45 (46.4)	
No	111 (57.2)	52 (53.6)	
Work During Pregnancy			0.803
Yes	103 (53.1)	50 (51.5)	
No	91 (46.9)	47 (48.5)	
Premature Birth			< 0.001^*^
Yes	0 (0.0)	29 (29.9)	
No	194 (100.0)	68 (70.1)	
Mode of Delivery			< 0.001^*^
Vaginal delivery	128 (66.0)	41 (42.3)	
Cesarean delivery	66 (34.0)	56 (57.7)	
Postpartum Blood Loss (mL)			0.005^*^
< 500	189 (97.4)	87 (89.7)	
≥500	5 (2.6)	10 (10.3)	
Newborn Gender			0.505
male	84 (43.3)	46 (47.4)	
female	110 (56.7)	51 (52.6)	
Maternal Blood V Concentration			0.011^*^
Low concentration (< 3.64 μg/L)	108 (55.67)	38 (39.18)	
High concentration (≥3.64 μg/L)	86 (44.33)	59 (60.82)	
Maternal Blood Pb Concentration			< 0.001^*^
Low concentration (< 86.70 μg/L)	146 (75.26)	52 (53.61)	
High concentration (≥86.70 μg/L)	48 (24.74)	45 (46.39)	

^*^Significant results, *P*-value < 0.05.

### 3.2 The association between maternal blood vanadium levels and CHDs in offspring

The maternal blood samples were classified into low and high V concentration groups based on a predetermined cut-off value: Low concentration (< 3.64μg/L) and High concentration (≥3.64μg/L). This categorization enabled a detailed analysis of the association between varying V levels and the risk of CHDs in offspring. As shown in Table 2, the aOR^2^ model reveals an increased association with CHDs (aOR^2^ = 2.15, 95% CI[1.26–3.66]; *p* = 0.005), including isolated CHDs (aOR^2^ = 2.30, 95% CI[1.11–4.79]; *p* = 0.025), multiple CHDs (aOR^2^ = 2.39, 95% CI[1.20–4.75]; *p* = 0.013), PDA (aOR^2^ = 1.99, 95% CI[1.09–3.64]; *p* = 0.025), and ASDs (aOR^2^ = 2.94, 95% CI[1.46–5.93]; *p* = 0.003), in the group with high maternal blood V concentrations. These findings should be interpreted as associations, rather than causal effects, suggesting that maternal blood V exposure may be associated with an elevated risk of CHDs in offspring.

**Table 2:** Risks for fetal CHDs in different vanadium in maternal blood concentrations.

**Group**	**Levels**	**Cases**	**Controls**	**aOR^1^ (95% CI)**	**aOR^2^ (95% CI)**
		***N*** = **97 (%)**	***N*** =**194 (%)**		
All CHDs	Low	38 (39.2)	108 (55.7)	1	1
	High	59 (60.8)	86 (44.3)	2.13 (1.27–3.58) ^*^	2.15 (1.26–3.66) ^*^
The isolated CHDs	Low	17 (17.5)	108 (55.7)	1	1
	High	26 (26.8)	86 (44.3)	2.20 (1.08–4.48) ^*^	2.30 (1.11–4.79) ^*^
The multiple CHDs	Low	17 (17.5)	108 (55.7)	1	1
	High	31 (32.0)	86 (44.3)	2.26 (1.15–4.41) ^*^	2.39 (1.20–4.75) ^*^
The Syndrome CHDs	Low	4 (4.1)	108 (55.7)	1	1
	High	2 (2.1)	86 (44.3)	0.81 (0.14–4.73)	0.79 (0.14–4.65)
PDA	Low	30 (30.9)	108 (55.7)	1	1
	High	40 (41.2)	86 (44.3)	1.86 (1.04–3.34) ^*^	1.99 (1.09–3.64) ^*^
ASDs	Low	15 (15.5)	108 (55.7)	1	1
	High	33 (34.0)	86 (44.3)	2.85 (1.42–5.70) ^*^	2.94 (1.46–5.93) ^*^
VSDs	Low	2 (2.1)	108 (55.7)	1	1
	High	5 (5.2)	86 (44.3)	3.51 (0.64–19.39)	3.94 (0.69–22.62)
AVSDs	Low	2 (2.1)	108 (55.7)	1	1
	High	2 (2.1)	86 (44.3)	1.34 (0.18–9.84)	1.58 (0.20–12.54)

aOR refers to the adjusted odds ratio. Logistic regression was employed to calculate the odds ratios and their corresponding 95% CIs. Based on the optimal cut-off value determined by the ROC curve analysis, V concentrations in the blood of pregnant women were categorized into high- and low-concentration groups. The aOR^1^ model was adjusted for preterm birth, mode of delivery, and postpartum blood loss. The aOR^2^ model further adjusted for Pb concentration based on the aOR^1^ model. Abbreviations: PDA, Patent Ductus Arteriosus; ASDs, Atrial Septal Defects; VSDs, Ventricular Septal Defects; AVSDs, Atrioventricular Septal Defects.^*^ Significant results, *P*-value < 0.05.

### 3.3 The association between maternal blood lead levels and CHDs in offspring

The maternal blood samples were classified into low and high Pb concentration groups based on a predetermined cut-off value: Low concentration (< 86.70 μg/L) and High concentration (≥86.70 μg/L). This categorization enabled a detailed analysis of the association between varying Pb levels and the risk of CHDs in offspring. As shown in Table 3, the aOR^2^ model reveals an increased association with CHDs (aOR^2^ = 2.76, 95% CI[1.61–4.70]; *p* < 0.001), including isolated CHDs (aOR^2^ = 3.05, 95% CI[1.48–6.26]; *p* = 0.002), multiple CHDs (aOR^2^ = 3.01, 95% CI[1.52–5.96]; *p* = 0.002), PDA (aOR^2^ = 2.98, 95% CI[1.64–5.42]; *p* < 0.001), ASDs (aOR^2^ = 2.04, 95% CI[1.01–4.12]; *p* = 0.048), and VSDs (aOR^2^ = 8.66, 95% CI[1.52–49.51]; *p* = 0.015) in the group with high maternal blood Pb concentrations. These findings should be interpreted as associations, rather than causal effects, suggesting that maternal blood Pb exposure may be associated with an elevated risk of CHDs in offspring.

**Table 3:** Risks for fetal CHDs in different lead in maternal blood concentrations.

**Group**	**Levels**	**Cases**	**Controls**	**aOR^1^ (95% CI)**	**aOR^2^ (95% CI)**
		***N*** = **97 (%)**	***N*** =**194 (%)**		
All CHDs	Low	52 (53.6)	146 (75.3)	1	1
	High	45 (46.4)	48 (24.7)	2.74 (1.62–4.64) ^*^	2.76 (1.61–4.70) ^*^
The isolated CHDs	Low	23 (23.7)	146 (75.3)	1	1
	High	20 (20.6)	48 (24.7)	2.93 (1.44–5.96) ^*^	3.05 (1.48–6.26) ^*^
The multiple CHDs	Low	25 (25.8)	146 (75.3)	1	1
	High	23 (23.7)	48 (24.7)	2.85 (1.46–5.55) ^*^	3.01 (1.52–5.96) ^*^
The Syndrome CHDs	Low	4 (4.1)	146 (75.3)	1	1
	High	2 (2.1)	48 (24.7)	1.25 (0.21–7.29)	1.28 (0.22–7.48)
PDA	Low	37 (38.1)	146 (75.3)	1	1
	High	33 (34.0)	48 (24.7)	2.85 (1.58–5.13) ^*^	2.98 (1.64–5.42) ^*^
ASDs	Low	30 (30.9)	146 (75.3)	1	1
	High	18 (18.6)	48 (24.7)	1.92 (0.97–3.79)	2.04 (1.01–4.12) ^*^
VSDs	Low	2 (2.1)	146 (75.3)	1	1
	High	5 (5.2)	48 (24.7)	7.85 (1.43–42.96) ^*^	8.66 (1.52–49.51) ^*^
AVSDs	Low	0 (0.0)	146 (75.3)	1	1
	High	4 (4.1)	48 (24.7)	–	–

aOR refers to the adjusted odds ratio. Logistic regression was employed to calculate the odds ratios and their corresponding 95% CIs. Based on the optimal cut-off value determined by the ROC curve analysis, Pb concentrations in the blood of pregnant women were categorized into high- and low-concentration groups. The aOR^1^ model was adjusted for preterm birth, mode of delivery, and postpartum blood loss. The aOR^2^ model further adjusted for V concentration based on the aOR^1^ model. Abbreviations: PDA, Patent Ductus Arteriosus; ASDs, Atrial Septal Defects; VSDs, Ventricular Septal Defects; AVSDs, Atrioventricular Septal Defects. ^*^ Significant results, *P*-value < 0.05.

### 3.4 . Interaction effects between vanadium and lead

As shown in Table 4, in the analysis of CHDs and their subtypes, no significant multiplicative or additive interaction effects between V and Pb were observed. However, the combined high levels of V and Pb in maternal blood were significantly associated with an increased risk of CHDs in offspring. Specifically, compared to the low V and low Pb level group, the high V and high Pb level group showed an increased risk of CHDs (aOR = 6.29, 95% CI [2.92–13.54]; *p* < 0.001), including the isolated CHDs (aOR = 7.08, 95% CI[2.55–19.65]; *p* < 0.001), the multiple CHDs (aOR = 7.18, 95% CI[2.67–19.27]; *p* < 0.001), PDA (aOR = 6.06, 95% CI[2.53–14.55]; *p* < 0.001), ASDs (aOR = 6.12, 95% CI[2.26–16.61]; *p* < 0.001), and VSDs (aOR = 20.54, 95% CI[1.94–216.9]; *p* = 0.012).

**Table 4:** Analyzes the effect of vanadium and lead interaction in maternal blood on offspring CHDs using multivariate logistic regression.

**Exposure**	**All CHDs**		**The isolated CHDs**		**The multiple CHDs**		**The Syndrome CHDs**	
	**Case/control (** ***n*** **)**	**aOR (95% CI)**	**Case/control (** ***n*** **)**	**aOR (95% CI)**	**Case/control (** ***n*** **)**	**aOR (95% CI)**	**Case/control (** ***n*** **)**	**aOR (95% CI)**
Low V and low Pb	23/79	1	10/79	1	9/79	1	4/79	1
Low V and high Pb	15/29	1.79 (0.81–3.96)	7/29	1.92 (0.64–5.80)	8/29	2.30 (0.80–6.61)	0/29	–
High V and low Pb	29/67	1.57 (0.81–3.04)	13/67	1.71 (0.68–4.33)	16/67	1.88 (0.77–4.63)	0/67	–
High V and high Pb	30/19	6.29 (2.92–13.54) ^*^	13/19	7.08 (2.55–19.65) ^*^	15/19	7.18 (2.67–19.27) ^*^	2/19	2.14 (0.35–13.00)
Multiplicative interaction		2.24 (0.76–6.64)		2.15 (0.50–9.15)		1.66 (0.41–6.67)		–
RERI		3.93 (−0.27–8.13)		4.44 (−1.66–10.53)		3.99 (−1.85–9.83)		3.14 (−0.72–6.99)
AP		0.63 (0.31–0.94) ^*^		0.63 (0.23–1.03) ^*^		0.56 (0.12–1.00) ^*^		1.47 (0.62–2.31) ^*^
SI		3.90 (1.01–15.13) ^*^		3.71 (0.73–18.96)		2.83 (0.72–11.18)		–
**Exposure**	**PDA**		**ASDs**		**VSDs**		**AVSDs**	
	**Case/control (** ***n*** **)**	**aOR (95% CI)**	**Case/control (** ***n*** **)**	**aOR (95% CI)**	**Case/control (** ***n*** **)**	**aOR (95% CI)**	**Case/control (** ***n*** **)**	**aOR (95% CI)**
Low V and low Pb	16/79	1	10/79	1	1/79	1	0/79	1
Low V and high Pb	14/29	2.42 (1.02–5.74) ^*^	5/29	1.33 (0.41–4.29)	1/29	2.88 (0.17–48.82)	2/29	–
High V and low Pb	21/67	1.65 (0.77–3.57)	20/67	2.30 (0.98–5.39)	1/67	1.26 (0.07–21.30)	0/67	–
High V and high Pb	19/19	6.06 (2.53–14.55) ^*^	13/19	6.12 (2.26–16.61) ^*^	4/19	20.54 (1.94–216.9) ^*^	2/19	–
Multiplicative interaction		1.52 (0.46–5.01)		2.00 (0.45–8.80)		5.67 (0.15–215.25)		–
RERI		2.99 (−1.53–7.51)		3.49 (−1.63–8.61)		17.40 (−24.52–59.33)		–
AP		0.49 (0.04–0.95) ^*^		0.57 (0.13–1.01) ^*^		0.85 (0.48–1.21) ^*^		0.37 (-0.94–1.67)
SI		2.44 (0.71–8.47)		3.14 (0.66–14.89)		9.15 (0.24–345.56)		1.58 (0.20–12.54)

aOR refers to the adjusted odds ratio. Logistic regression was employed to calculate the odds ratios and their corresponding 95% CIs. The aOR model was adjusted for preterm birth, mode of delivery, and postpartum blood loss. Abbreviations: PDA, Patent Ductus Arteriosus; ASDs, Atrial Septal Defects; VSDs, Ventricular Septal Defects; AVSDs, Atrioventricular Septal Defects; RERI, Relative Excess Risk due to Interaction; AP, Attributable Proportion due to Interaction; SI, Synergy Index. ^*^ Significant results, *P*-value < 0.05.

## 4 Discussion

The objective of this study was to examine the potential interaction between maternal exposure to V and Pb during pregnancy and the risk of CHDs in offspring. While no significant interaction was found between maternal blood levels of V and Pb and the overall incidence of CHDs, this study—being the first to investigate the association between maternal V exposure during pregnancy and offspring CHDs risk—revealed a significant relationship between individual exposure to V and Pb and an increased risk of CHDs in offspring. Furthermore, the results showed that pregnant women with elevated levels of V and Pb in their blood had a markedly higher risk of having offspring with CHDs compared to those with lower levels of these metals. To facilitate risk stratification and result interpretation, we used ROC analysis to determine the grouping cutoff. This cutoff should not be interpreted as a causal threshold or a clinically recommended reference value. In parallel, questionnaire-derived covariates were collected to capture key sociodemographic and obstetric characteristics that could confound the associations between maternal metal exposure and CHDs risk. Consistent with the matched design, most baseline characteristics were well balanced between cases and controls (Table 1), and differences in preterm birth, delivery mode, and postpartum blood loss were further adjusted for in multivariable models to minimize residual confounding.

To the best of our knowledge, this is the first epidemiological study to examine associations between prenatal V exposure during late pregnancy and CHDs in offspring. Utilizing a prospective longitudinal study design, we provide evidence indicating that prenatal exposure to V may be linked to an elevated risk of CHDs in offspring, including several key subtypes. Although the precise biological mechanisms linking prenatal V exposure to CHDs in offspring remain unclear, several plausible hypotheses have been proposed to explain its toxic effects. V primarily enters the maternal body through the digestive and respiratory tracts, crosses the placental barrier into the fetal environment, and accumulates in the fetus (33, 34). Studies in both animals and humans have demonstrated that V exposure increases oxidative stress, primarily by generating reactive oxygen species (ROS), which primarily manifest as protein oxidation, DNA damage, and lipid peroxidation (35–37). Excessive ROS may contribute to the development of CHDs by disrupting both intercellular and intracellular signaling pathways (38). Furthermore, research has shown that ROS can trigger apoptosis through the p53-mitochondria-caspase pathway, resulting in embryotoxicity (39).

Our findings suggest that elevated Pb levels in maternal blood are associated with an increased risk of CHDs, consistent with previous research (26, 40–42). Compared to previous studies (26, 40–42), our research offers a more detailed classification of CHDs, considering both the severity of the defects and incorporating ICD-10 codes for a comprehensive subtype analysis. Pb can cross the placental barrier, transferring from the mother to the fetus, with fetal blood Pb levels positively correlated with maternal blood Pb levels (43). The mechanisms through which Pb contributes to CHDs remain incompletely understood, but several pathways have been proposed to explain its toxic effects on embryonic heart development. First, elevated blood Pb levels reduce the activity of superoxide dismutase, leading to oxidative stress. This oxidative stress can result in cellular damage or death, increased lipid and protein peroxidation, DNA damage, impaired cardiac remodeling, and other cardiovascular complications (40). Additionally, studies suggest that Pb exposure may contribute to CHDs by disrupting DNA methylation (44, 45). Previous research has shown that prenatal Pb exposure can induce changes in genomic DNA methylation in umbilical cord blood (46, 47).

Our study is the first to examine the interaction between V and Pb in relation to CHDs. Although we did not observe statistically significant additive or multiplicative interactions between V and Pb concerning CHDs, pregnant women with elevated blood levels of both metals exhibited a significantly higher risk of having offspring with CHDs compared to those with lower levels. Furthermore, an intriguing pattern emerged in Tables 2, 3. After adjusting the ORs, the OR values in the aOR^2^ were slightly higher than those in aOR^1^. Although the increase was modest, this finding, coupled with the strong positive correlation between maternal blood concentrations of V and Pb depicted (*r* = 0.9095, *p* < 0.001), provides reasonable evidence suggesting a potential synergistic effect of these metals in the development of CHDs. In the previous discussion, we highlighted that prior studies have identified oxidative stress as a potential mechanism through which both V and Pb may contribute to CHDs. This finding offers a valuable direction for future research into the molecular mechanisms underlying the synergistic effects of these metals in CHDs development.

This study is based on the prospective Lanzhou Birth Cohort, which includes 10,823 participants, providing a robust foundation for our research. Furthermore, the case and control groups were carefully matched by age and residential area, minimizing the potential influence of these factors on V and Pb exposure. However, several limitations must be acknowledged. First, maternal blood samples were collected during the third trimester of pregnancy, which may not accurately reflect the association between V and Pb exposure during early pregnancy and CHDs. Second, while this study assessed the relationship between prenatal V and Pb exposure and offspring CHDs from a population-based perspective and suggested a potential synergistic effect, further research is necessary to explore the metabolic dynamics and pathogenic mechanisms underlying CHDs. Considering that, in addition to V and Pb, metals such as arsenic, cadmium, and mercury have also been associated with CHDs in some studies (8), this indicates that exposure to other metals may synergistically interact with V and Pb to collectively influence fetal cardiac development. Future work will apply multi-metal mixture models (e.g., BKMR/WQS) to identify dominant contributors among co-occurring metal exposures. Third, the ROC-derived cutoff may be influenced by the specific characteristics of the present study sample and therefore requires external validation of its stability and generalizability in independent populations. Finally, due to the specific industrial background and environmental factors in Lanzhou, the study findings may differ in other regions. Therefore, future research should take into account the population characteristics and environmental contexts of different areas.

## 5 Conclusion

Overall, our study revealed a significant association between maternal whole blood levels of V and Pb in late pregnancy and an increased risk of CHDs in newborns. This association was evident for both individual and combined exposures. Furthermore, we observed a potential synergistic effect of maternal V and Pb concentrations on the occurrence of total CHDs. However, because the measured concentrations reflect late-pregnancy levels rather than exposure during the critical early embryonic period of cardiac morphogenesis, a direct causal inference regarding early fetal heart development cannot be established. These findings warrant replication in larger cohorts and further mechanistic investigation into the role of prenatal V and Pb exposure in the development of congenital heart defects in offspring.

## Data Availability

The raw data supporting the conclusions of this article will be made available by the authors, without undue reservation. Inquiries for access to the datasets should be directed to 1038817191@qq.com.
